# ^99m^Tc-DTPA Diuretic Renography with 3 hours late output fraction in the evaluation of hydronephrosis in children

**DOI:** 10.1590/S1677-5538.IBJU.2017.0131

**Published:** 2018

**Authors:** Carlos J. R. Simal

**Affiliations:** 1 Departamento de Anatomia e Imagem, Faculdade de Medicina da Universidade Federal de Minas Gerais, Belo Horizonte, Brasil; 2 Divisão de Medicina Nuclear, Hospital Felicio Rocho, Belo Horizonte, Minas Gerais, Brasil

**Keywords:** Hydronephrosis, Ureteral Obstruction, Cakut [Supplementary Concept], Technetium Tc 99m Pentetate

## Abstract

**Objective:**

Dynamic renal scintigraphy complemented by late gravity assisted postvoid images to 60 minutes is a frequently used diagnostic test in the evaluation of hydrone- phrosis. The objective of this study is to evaluate the effectiveness in acquiring images at 180 minutes to calculate the late output fraction (LOF) of ^99m^Tc-DTPA in the diagno- sis of ureteropelvic junction obstruction (UPJO).

**Materials and Methods:**

A retrospective study of 177 patients (196 renal units) of sus- pected cases of clinical UPJO was conducted. The patients were submitted to at least two dynamic renal scintigraphies of ^99m^Tc-DTPA, with the addition of furosemide (F0), with a mean age of 4.3±3.8 years for the first study, and a follow-up of 2.7±2.5 years.

**Results:**

For diagnosis based on renal curves, a 100% sensitivity, 82.2% specificity, positive predictive value (PPV) of 10.4% and negative predictive value (NPV) of 100% were estimated. For diagnosis based on LOF, a 100% sensitivity, 96.3% specificity, PPV of 35.7% and NPV of 100% were estimated.

**Conclusion:**

A LOF <10% is indicative of UPJO, and a LOF ≥15% is indicative of no UPJO. The data demonstrate that LOF presents equivalent sensitivity and NPV, and higher specificity and PPV in comparison to diagnosis based on renal curves, and is useful in the evaluation and follow-up of suspected cases of UPJO.

## INTRODUCTION

The routine use of fetal ultrasound (US) is an important resource in the early detection and treatment of hydronephrosis caused by ureteropel-vic junction obstruction (UPJO). The incidence of hydronephrosis in fetal and neonatal ultrasound is on the order of 1 to 5% and may be caused by various conditions such as vesico-ureteral reflux, obstructions to the urinary ducts and transitory dilation of the renal calyces, pelvis or ureters (1). The challenge created by the detection of antenatal hydronephrosis (ANH) is the correct identification of effectively obstructed cases (10 to 30%), as opposed to transitory hydronephrosis cases (41 to 88%), where surgery should be avoided (1).

The evaluation of patients with ANH in general is based upon pelvic dimensions determined through ultrasound, complemented by micturating cystourethrogram and renal function evaluation through renal scintigraphies dynamic (DYN) with ^99m^Tc-DTPA or ^99m^Tc-MAG3 and static with ^99m^Tc-DMSA.

Although the analysis of the renogram is the most usual in the evaluation of the capacity to eliminate radiopharmaceuticals filtered by the kidneys, especially T_1/2_, other approaches have been proposed, such as normalized residual activity (NORA), output efficiency (OE), pelvic excretion efficiency (PEE), and delayed images (2–7).

The objective of the present study is to analyze the applicability of late output fraction (LOF) in images acquired 3 hours after the administration of ^99m^Tc-DTPA, in the evaluation of UPJO.

## MATERIALS AND METHODS

The study has been approved by the Felicio Rocho Hospital's review board and the need for informed consent was waived. In the period between November 12th, 1997 and June 16th, 2014, 7,359 DYN were performed. The criteria for inclusion in the study were: at least two dynamic and static renal scintigraphy (pre and post-treatment), absence of other abnormalities such as duplication of excretion system, vesico-ureteral reflux, posterior ureteral valve, uretero-vesical junction obstruction and multicystic dysplastic kidney. 177 patients suspected to suffer from UPJO were included in the retrospective study. 58.8% (104/177) were male, and 41.2% (73/177) were female, constituting a total of 346 renal units (8 unilateral nephrectomies at first scan). Of the total number of renal units, 56.6% (196/346) were suspect for UPJO. Of these, 19.1% (66/346) units at right, and 25.4% (88/346) units at left side. In 21 patients, compromise was bilateral, corresponding to 12.1% (42/346) of the renal units. The mean ages were 4.3±3.8 years for the first, and 7.0±4.8 years for the second scan, with a follow-up of 2.7±2.5 years.

Relative renal function was evaluated by scintigraphy with ^99m^Tc-DMSA, at a dosage of 37-74 MBq (1-2 mCi).

**Figure 1:**
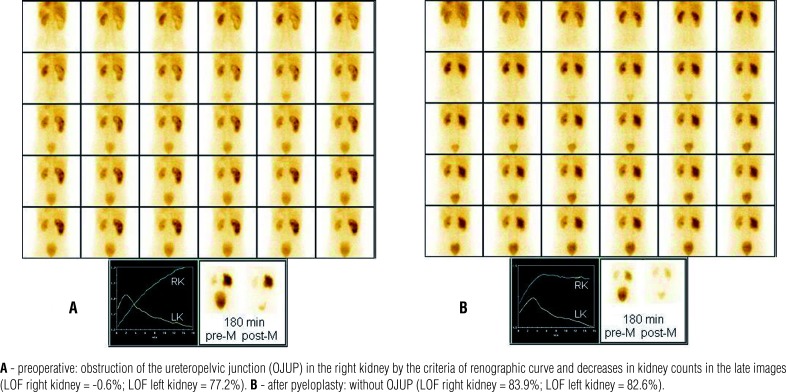
Dynamic renal scintigraphy with 99mTc-DTPA with furosemide administration (F0) and late (180 minutes) premicturition (pre-M) and gravity assisted postmicturition (post-M) images.

The DYN1 and DYN2 (the first and second dynamic renal scintigraphies, respectively) were acquired during a period of 20 minutes immediately after the intravenous injection of 74-185 MBq (2-5 mCi) of ^99m^Tc-DTPA, with simultaneous administration of furosemide (1mg/kg of weight). Three hours after the administration of the radiopharmaceuticals, late pre and postvoid images were acquired (Figure-1). In the acquisition of the postvoid images, patients were maintained in an orthostatic position for 5 minutes, in order to contribute to renal emptying.

The patients were orally hydrated 1 hour before the administration of the radiopharmaceuticals, with two or three cups of water for older children, and breastfeeding for infants. When possible, patients were requested to empty their bladders before beginning the scan. After the dynamic phase, the patients were kept hydrated for two hours more, through means of water intake (two or three cups) or breastfeeding. Older children were oriented to avoid micturition for 1 hour before the late images. The children without sphincter control, when presented for the 3 hour late images with bladder already empty, had acquired a “pre-void” image. They were maintained in standing position for 5 minutes, and following this, the “post-void” image was acquired.

The images were analyzed visually for the detection of any retention of the tracer in the renal calyces, pelvis or ureters, and renographic curves were drawn. Renal regions of interest (ROIs) were delineated in the last image of the dynamic sequence (20 minutes), and these ROIs were replicated in the late images both pre and postvoid, in order to estimate the radioactive renal activity.

The elimination phase in the renographic curves (phase 3) were initially classified from 0 to 6 (0 = adequate drainage, 1 = light fall in drainage, 2 = moderate fall in drainage, 3 = accentuated fall in drainage, 4 = practical absence of drainage, 5 = absence of drainage, 6 = accentuated hypofunction). For statistical analysis, the classification of renographic curves was summarized as follows: curves of type 0 to 3 were considered as non-obstructive and curves of type 4 and 5 as obstructive. Curves of type 6 did not permit the differentiation between non-obstructive and obstructive, and were thus considered not possible to be analyzed.

The fraction of elimination of the radiopharmaceutical in each renal unit was calculated using the equation: LOF (%) = (counts at 20 minutes – counts at 180 minutes) x 100 / counts at 20 minutes. For statistical analysis, obstruction was defined as a LOF of ≤12.4%, independent of the renographic standard.

Upon return for the second nuclear medicine evaluation, data was collected on the evolution of the patient, in particular the impression of the physician about the obstructive process and the treatment method chosen, clinical or surgical.

## RESULTS

The distribution of scintigraphic diagnosis based upon evaluation of renal curves, for the first (CURVE 1) and the second (CURVE 2) studies, and upon renal counts at 20 minutes along with 180 minutes images for the first (LOF 1) and second (LOF2) studies, is presented in Table-1.

**Table 1:** Scintigraphic diagnosis.

Scan	Diagnostic criterion	Scintigraphic diagnosis
		Unobstructed 65.9% (228/346)
	CURVE1	Obstructed 32.1% (111/346)
		Hypofunctioning* 2.0% (7/346)
**First**		Unobstructed 84.1% (291/346)
	LOF1 ‡	Obstructed 15.9% (55/346)
		Unobstructed 80.9% (279/345)
	CURVE2	Obstructed 17.7% (61/345)
		Hypofunctioning* 1.4% (5/345)
**Second** †		Unobstructed 96.2% (332/345)
	LOF2‡	Obstructed 3.8% (13/345)

**CURVE1 and 2:** renal curves in the first and second scans.

**LOF1 and 2:** output fraction in the first and second scans.

* severely hypofunctioning kidneys not allowing differentiation by curve analysis between obstruction and non obstruction.

† one unilateral nephrectomy between the first and second scintigraphy.

‡ a LOF ≤12.4% was considered as obstructive.

The analysis of CURVE 1 and LOF 1, in relation to the distribution by sex and affected side (Table-2) shows a higher incidence of obstructive process on the left side (Pearson Chi-Square p<0.05).

**Table 2:** Distribution of diagnosis of the obstructive type for affected side and sex.

Diagnostic criterion	Side*	Sex	Renal units
	Right 39.6% (44/111)	Boys	59.1% (26/44)
CURVE1 32.7% (111/339)		Girls	40.9% (18/44)
	Left 60.4% (67/111)	Boys	68.7% (46/67)
		Girls	31.3% (21/67)
	Right 36.4% (20/55)	Boys	50.0% (10/20)
LOFI† 15.9% (55/346)		Girls	50.0% (10/20)
	Left 63.6% (35/55)	Boys	65.7% (23/35)
		Girls	34.3% (12/35)

**CURVE1:** renal curves in the first scan.

**LOF1:** output fraction in the first scan.

* Pearson Chi-Square p< 0.05

† a LOF ≤12.4% was considered as obstructive.

Clinical treatment was initiated in 71.1% (246/346), and surgical treatment instituted in 28.9% (100/346) of the renal units. Without considering contralateral renal units not suspect for obstruction, and thus considering only 196 renal units indicating clinical obstruction and renal curves with at least a moderate degree of delay in excretion, clinical treatment was the choice instituted in 52.6% (103/196) of cases, and pieloplasty in 46.9% (92/196) of cases. One kidney (0.5% - 1/196) with scintigraphic diagnosis of partial urine flow obstruction, and 22.1% of relative function in renal static scintigraphy, was nephrectomized. 2.0% (7/346) renal units were submitted to pielo-plasty based upon suspect on clinical examination, without a scintigraphic diagnosis of obstruction. Table-3 shows the scintigraphic evaluations and treatments of the totality of the renal units studied. Measures of agreement Kappa of 0.428 (p<0.001) for CURVE 1 *versus* LOF 1, and of 0.221 (p<0.001) for CURVE 2 *versus* LOF 2 were found. In the comparison of DYN 1 and DYN 2, only for those patients submitted to conservative clinical treatment, measures of agreement Kappa of 0.158 (p<0.001) for CURVE1 *versus* LOF2, and of 0.512 (p<0.001) for (LOF1) *versus* LOF2 were found.

**Table 3:** Pre and post-treatment scintigraphic evaluation in all renal units studied.

Diagnostic criterion (pretreatment)	Scintigraphic diagnosis (pretreatment)	Treatment	Diagnostic criterion (post-treatment)	Scintigraphic diagnosis (post-treatment)
	Unobstructed 57.2% (198/346)			Unobstructed 100% (198/198)
CURVE1	Obstructed 13.9% (48/346)		LOF2*	Obstructed 10.4% (5/48)
		Clinical		
	Unobstructed 67.1% (232/346)			Unobstructed 100% (232/232)
LOF1*	Obstructed 4.0% (14/346)		LOF2*	Obstructed 35.7% (5/14)
	Unobstructed 8.7% (30/346)			Unobstructed 100% (30/30)
CURVE1	Obstructed 20.2% (70/346)		LOF2*	Obstructed 11.4% (8/70)†
LOF1*	Unobstructed 17.1% (59/346)	Pyeloplasty		Unobstructed 89.8% (53/59)
	Obstructed 11.8% (41/346)		LOF2*	Obstructed 4.9% (2/41)†

**CURVE1:** renal curves in the first scan.

**LOF1 and 2:** late output fraction in the first and second scans.

* a LOF ≤12.4% was considered as obstructive.

† 40 renal units undergoing pyeloplasty and one nephrectomy.

For the diagnosis based upon CURVE 1, a sensitivity of 100%, a specificity of 82.2%, a positive predictive value (PPV) of 10.4%, and a negative predictive value (NPV) of 100% were es-timated. For the diagnosis based upon LOF1, a sensitivity of 100%, a specificity of 96.3%, a PPV of 35.7%, and a NPV of 100% were estimated.

The study found 21.4% (74/346) renal units with ^99m^Tc-DMSA relative function below 40.0%, and 78.6% (272/346) above 40.1%. Of the 74 units with relative function below 40.0%, 51.4% (38/74) were submitted to clinical treatment and 48.6% (36/74) to surgical treatment, while of the 272 units with relative function above 40.1%, 69.9% (190/272) were submitted to clinical treatment and 30.1% (82/272) to surgical treatment (p>0.05).

The therapeutic choice, clinical or surgical, and its relationship with scintigraphic diagnosis of obstruction *versus* non-obstruction is presented in Table-4.

**Table 4:** Relationship between the scintigraphic diagnosis and treatment established in clinical suspicion of UPJO.

Scintigraphic criteria and diagnosis (pretreatment)		Treatment	
CURVE1	LOF1*	Clinical	Pyeloplasty
Unobstructed	Unobstructed	88.6% (195/220)	11.4% (25/220)
Obstructed	Obstructed	23.4% (11/47)	76.6% (36/47)
Unobstructed	Obstructed	37.5% (3/8)	62.5% (5/8)
Obstructed	Unobstructed	52.1% (37/71)	47.9% (34/71)

**CURVE1:** renal curves in the first scan.

**LOF1:** output fraction in the first scan.

* a LOF ≤12.4% was considered as obstructive.

## DISCUSSION

The distinction between transitory dilation of drainage ducts and of UPJO is somewhat complex, and approaches vary widely among institutions. No study is considered to be a gold standard in the evaluation of obstructive renal processes (1–2).

Though the analysis of renal curves, including the calculation of T_1/2_, and the use of other indicators (OE and NORA) (6, 8) are the most common approaches, the acquisition of late pre and postvoid gravity assisted images have been considered useful (3, 7, 9, 10). T_1/2_ is no longer considered sufficient in the evaluation of renal drainage (10–12). Frequently, late pre and postvoid images contradict curves indicating obstructive pattern, and also supply more consistent and reproducible data than renogram (2, 13). This may explain the relatively low correlation found between renal curves and LOF by the Kappa agreement analysis.

LOF is an attempt to evaluate renal draining considering renal counts at 20 minutes and at 3 hours pre and postvoid images after ^99m^Tc-DTPA and furosemide injections. For OE and NORA the ideal length of time for the realization of late images varies with MTT (6). For smaller MTT, pre and postvoid images at 60 minutes are probably sufficient, while for larger MTT, later images might be necessary. Late images at 60 and 120 minutes were attempted to calculate LOF before 180 minutes was considered more reliable. LOF incorporates the additional advantages of postmicturition images replacing bladder catheterization (2, 3). OE has less dependence on the level of renal function (2) and LOF seems to work well even in cases of reduced renal function.

The cutoff point which characterizes obstruction *versus* non-obstruction, by means of LOF, was defined as experience was gathered. A LOF of up to 10% is strongly indicative of UPJO, while a LOF greater than or equal to 15% is indicative of a non-obstructive process, with >10% and <15% being in the range of uncertainty. In the present study, for effects of statistical analysis, values of ≤12.4% were used as indicators of urinary flow obstruction. In practice, the criteria for defining a diagnosis as obstructive has been LOF ≤10%, and to disregard obstruction, LOF ≥25%. For the values >10% e <25%, a new study is recommended after 6 months for the sake of certainty.

In the estimation of diagnostic capacity of the criteria utilized in the present study, the second dynamic renal scintigraphy of the group submitted to clinical treatment was used. Both clinical impression and the results obtained through LOF2 were, together, considered to be the true diagnosis for the definition of obstruction *versus* non-obstruction. After a mean follow-up time of 2.7±2.5 years, if an unobstructive diagnosis by DYN1 were in error, a significant deterioration in renal function would have been expected, which did not occur. As such, it is reasonable to think that the DYN1 diagnosis was correct. The option to utilize LOF2 and not CURVE2 was due to the apparently better discrimination of obstruction *versus* non-obstruction permitted through LOF, compared to renal curves, as has already been observed with late pre and postvoid images (2, 13).

In the group submitted to clinical treatment, the rate of non-obstructive diagnosis was 57.2% (198/346) for CURVE1 and 67.1% (232/346) for LOF1. These diagnosis proved true in DYN2, with 100% of non-obstruction for both groups, that is, a larger number of non-obstructions through LOF1 criteria when the natural evolution of the cases is considered as a true result.

Only 10.4% (5/48) of cases diagnosed as obstructive through renal curves, and 35.7% (5/14) of cases diagnosed as obstructive through LOF, which were submitted to clinical treatment, presented signs of obstruction at second study. This data, in addition to demonstrating the higher specificity of diagnosis based upon LOF, appear to reflect that hydronephrosis caused by UPJO is a mutable process through time, possibly more functional than anatomical, and may thus cure spontaneously (13–18).

When considering the group of 220 renal units with very clear scintigraphic diagnosis, that is, curves and LOF frankly non-obstructive, it was observed that 11.4% (25/220) were submitted to pyeloplasty. On the other hand, in the group of 47 renal units with scintigraphic diagnosis, curves and LOF clearly obstructive, it was observed that 23.4% (11/47) were submitted to clinical tre-atment. No statistically significant difference was encountered in the choice of treatment as a result of relative renal function, estimated through ^99m^Tc--DMSA, below or above 40.0%. These data reflect the current lack of a standard in the choice of conduct (1, 2, 13). 10.2% (6/59) of renal units with diagnosis non-obstructive through LOF, submitted to surgical treatment, evolved into an obstructive pattern, suggesting that they were damaged by an inadequate intervention.

Further studies are necessary in order to identify which kidneys with detected ANH are at risk for deterioration in their function, which will permit more certainty in the choice of treatment. In any case, from the data presented, the diagnosis of urinary drainage obstruction based on LOF was superior to diagnosis based upon renal curves, and provided higher certainty in the indication of conservative treatment in about 17.2% of the patients whose LOF indicated absence of obstruction while renal curve suggested an obstructive process.

## CONCLUSIONS

This study to evaluate urinary flow obstruction utilizing dynamic renal scintigraphy with ^99m^Tc-DTPA demonstrated that an LOF of up to 10% is indicative of UPJO, while an LOF equal to or above 15% is not indicative of an obstructive process. The data showed that LOF provides equivalent sensitivity and NPV and higher specificity and PPV compared with renal curve based diagnosis, and is useful in the evaluation and follow-up of suspected cases of UPJO.
